# Rethinking Alcohol Harm Reduction

**DOI:** 10.1111/dar.70152

**Published:** 2026-03-31

**Authors:** Gabriel Caluzzi

**Affiliations:** ^1^ Centre for Alcohol Policy Research La Trobe University Melbourne Australia

**Keywords:** alcohol, alcohol harm reduction, alcohol policy, harm reduction

## Abstract

Alcohol harm reduction (AHR) includes a pragmatic set of strategies to reduce the acute harms of alcohol consumption without necessarily reducing use, such as managed alcohol plans and intoxication management facilities. While well established in illicit drug research, harm reduction's application to alcohol remains less explicitly examined, both conceptually and in terms of policy articulation. In contrast to population‐level consumption‐reduction strategies – many of which are effective at preventing harm – AHR often places greater emphasis on acute and immediate harms and may require consumers to take an active role, a feature that has raised concerns about industry mobilising ‘responsible drinking’ narratives. AHR also tends to be subsumed within other alcohol policies and has a less consolidated evidence base. However, drawing on international and Australian evidence, this article outlines practical and ethical reasons for a more explicit focus on AHR, including the substantial burden alcohol places on frontline health systems, its disproportionate impacts on socially disadvantaged populations and its frequent involvement in polydrug use and overdose. AHR can also facilitate cross‐sectoral collaboration and lateral policymaking, and contribute to the ethical obligation of policymakers to redress the harms of alcohol commercialisation. Beyond formal policy, AHR already occurs in clinical, educational and nightlife settings. Recognising this, future research and policy efforts should focus on clarifying how harm reduction can be more explicitly integrated within alcohol policy frameworks to complement population‐level approaches.

## 1 Introduction

Alcohol is associated with pleasure and sociality [1, 2, 3] and many people who consume alcohol do so at low risk of acute harms [4]. At the same time, alcohol‐related harm remains a significant public health issue, including its contributions to violence, accidents and disease. In Australia, this includes 1742 alcohol‐induced deaths and 4981 alcohol‐related deaths (such as alcohol attributable road accidents) in 2022 [4], and 4.1% of the total burden of disease [5]. Due in part to its widespread availability and social acceptance, Australian drug and alcohol professionals have ranked alcohol as the most harmful drug in terms of the overall health, social and economic burden it places on consumers and others [6].

Harm reduction is characterised by multiple traditions – as a pragmatic approach to risk reduction and a social justice movement – both of which focus on reducing the negative effects of substance use without necessarily requiring abstinence or reduced consumption [7, 8]. Originally focused on preventing HIV and blood‐borne viruses in drug use by supplying safe injecting equipment and encouraging safer injecting practices, it expanded to include other immediate harms experienced by people who use licit and illicit drugs [8]. While harm reduction approaches remain controversial against the backdrop of prohibition, they have also become a central feature of drug policy in many countries. For example, Australia's National Drug Strategy is grounded in a three‐pillar framework: supply reduction, demand reduction and harm reduction [9] – although in practice harm reduction receives little funding relative to the other pillars [10]. Harm reduction strategies aim at immediate, practical goals such as reducing drug‐related injuries, unsafe consumption practices and the spread of disease. They can also overlap with supply‐ and demand‐reduction strategies; for example, drug‐checking services are primarily a harm‐reduction approach aimed at reducing unsafe consumption of illicit drugs, but they may also reduce demand through drug information and contact with healthcare professionals.

Harm reduction has historically been more associated with illicit drugs than alcohol, largely because it emerged in response to harms produced by prohibitionist regulation, including criminalisation, black markets and stigma. While harm reduction cannot be rigidly applied to alcohol and illicit drugs in the same way, some of these issues are still relevant for alcohol, particularly in the context of marginalisation. For example, historic and ongoing issues around the criminalisation of public intoxication in Australia [11] highlight the complex needs and lived realities of disadvantaged people who drink. Indeed, there is a range of individual, peer‐based and structural alcohol harm reduction (AHR) interventions, such as managed alcohol programs, intoxication management facilities and targeted road safety interventions.

In this article, AHR is understood as a set of pragmatic and ethically grounded strategies that aim to reduce alcohol‐related harm. These include approaches that do not require abstinence, but that may support people to reduce consumption to manage immediate harms. While AHR exists in a range of modalities, I suggest that it is not always explicitly articulated and coherently mobilised in policy – particularly in contrast to other population‐level consumption‐reduction measures that are often packaged together as a policy suite [12]. This has led to it being under‐prioritised in alcohol policy.

## 2 Why AHR Has Been Underutilised?

There are several reasons why AHR may have been underutilised in policy. One is the concern around how it might be mobilised to individualise responsibility for alcohol‐related harm, undermining both harm reduction's social justice foundations and public health messaging. Industry actors may capitalise on ambiguous framings of AHR and consumer engagement to promote personal responsibility as a form of harm reduction, deflecting attention from commercial practices that produce harm [13, 14] and focusing only on narrow ‘problem’ minorities, such as young people or those in socio‐economically disadvantaged areas [15, 16]. For example, alcohol industry campaigns designed to minimise the harm from drinking tend to promote vague notions of ‘responsible drinking’, problematically framing AHR as a matter of self‐regulation, while resulting in little actual attitude or behaviour change [14, 16].

AHR also has a distinct functionality within alcohol policy that is not well articulated. The dose relationship between alcohol and long‐term harms [17] has led to a favouring of universal consumption‐reduction strategies such as taxation, advertising bans and restricted trading hours – measures which are effective in preventing harm at the population level [12]. In contrast, AHR initiatives are often targeted, selectively focusing on a narrow range of harms that may not be alleviated through population‐level approaches [18]. In this sense, population‐level policies primarily function as upstream prevention tools, whereas AHR is oriented towards mitigating immediate harms amongst those who may already be experiencing the ‘pointy end’ of harm.

Another issue is the problem of attribution. AHR is often conflated with consumption‐reduction initiatives. For example, drink–driving interventions designed to minimise alcohol‐related harm may reduce consumption, education campaigns often pursue both harm‐ and demand‐reduction, and the growth of pharmaceutical and cannabis substitution programs is intended to reduce harm and alcohol consumption in tandem [18, 19]. Similarly, measures to reduce consumption also reduce harm. Many of the World Health Organization's ‘SAFER’ initiatives designed to reduce alcohol‐related harms are explicitly focused on reducing consumption (e.g., taxation, reduced availability) [20]. While harm reduction and consumption‐reduction strategies are often complementary, alcohol policy has largely framed harm through population‐level consumption‐reduction approaches [12]. These approaches aim to reduce alcohol‐related harms and consumption, but are rarely conceptualised as harm reduction in the sense developed within drug policy. This risks overlooking the distinct objectives and potential benefits associated with harm reduction (e.g., improving service access, a focus on marginalised groups, safer consumption practices).

Finally, while the evidence base for effective AHR interventions is growing [18], it remains less extensive and less consolidated than other areas of alcohol policy research, such as taxation and availability restriction [12], and is often more challenging to mobilise as a policy lever. Policymakers operate under resource, political and time constraints [21], meaning simple, scalable policies are likely to be favoured. While this presents a practical barrier to policy implementation, it does not invalidate harm reduction as a framework for addressing alcohol. Rather, it offers an avenue for further research and examination to understand how and why AHR can be strategically and effectively utilised. As I argue, alcohol policy may more comprehensively reduce harm if the concept of harm reduction is explicitly considered.

## 3 Why AHR Deserves Renewed Attention in Policy Frameworks

Below, I outline several reasons why AHR warrants renewed policy attention. First, alcohol places a huge morbidity and mortality burden and places a significant burden on frontline healthcare systems. In Australia, it is responsible for 59% of drug‐related hospitalisations and 25% of all road injuries [4]. Alcohol‐related presentations at emergency departments have detrimental impacts on wait times and staff workloads, and can produce further harm [22]. AHR strategies can reduce this burden. Roadside breath‐testing has significantly reduced traffic injuries [23]. Intoxication management centres (or sobering up centres) that divert people away from hospitals can also reduce the load placed on health services and are popular with both frontline staff and patients [24]. From a pragmatic, practical and economic perspective, AHR has the capacity to be a useful strategy alongside primary prevention approaches to reduce emergency healthcare burden.

Second, alcohol‐related harm often intersects with other forms of social disadvantage, meaning marginalised populations bear greater exposure to alcohol‐related illness, injury and social harm [25, 26]. Population‐wide interventions, including taxation and minimum unit pricing, can reduce alcohol‐related harm at scale and may contribute to addressing socioeconomic inequalities in the long term [27]. However, individuals experiencing alcohol‐related harms alongside housing insecurity or mental illness may benefit less from these approaches, as their alcohol use often reflects complex needs that require tailored interventions [27, 28]. Here, AHR offers more immediate strategies to reach, support and assist with social service (re)entry. Intoxication management centres and mobile outreach patrols are examples of harm reduction efforts aimed at minimising acute harm while connecting people to support services [9]. Managed alcohol programs offer another pathway for individuals with chronic alcohol dependence and insecure housing. These programs provide measured alcohol doses to reduce withdrawal, prevent consumption of unsafe non‐beverage alcohol and facilitate housing and healthcare access [28, 29]. These approaches are not novel, but have yet to be consistently embedded within mainstream alcohol policy.

Third, harmful interactions between alcohol and other substances highlight the need to recognise the spillover effect alcohol has for people who consume alcohol and other drugs simultaneously. While alcohol is often overlooked as an element in polydrug use [30], it can heighten the risk of adverse drug interactions, overdose, impaired judgement and injury when combined with other psychoactive substances. For example, in British Columbia, alcohol has been present in 20%–29% of unregulated drug overdose deaths each year from 2014 to 2023 [31]. From an AHR perspective, overlooking alcohol's role in these interactions masks its contribution to overdose risk and reinforces the siloing of alcohol policy and other drug harm reduction frameworks.

Fourth, harm reduction cuts across various health, social, criminal, education and civil systems, and empowers ‘non‐elite’ actors (e.g., consumers and peer workers) to participate in and mobilise policy, particularly at the local level [32]. This invites collaboration, innovation and lateral policymaking that can address policy gaps and harms that may lie outside any single policy domain. Nightlife safety interventions that co‐ordinate in‐venue safety initiatives (e.g., staff training and crowd management) with external transportation options have been shown to reduce intoxication‐related injuries [33, 34]. Managed alcohol programs, intoxication management centres and public intoxication decriminalisation enable improved co‐ordination between healthcare, social services and police. Grassroots programs like the Vancouver Alcohol Strategy – a community‐led initiative integrating a suite of these initiatives [35] – show how collaborative AHR policies can build reach and responsiveness into broader public health strategies.

Finally, because many of the harms of alcohol can be attributed to legal regulatory frameworks that allow its purchase and consumption [12], harm reduction places a distinct ethical responsibility on governments to reduce harms through policies and interventions. Just as harm reduction emerged from ethical and social justice concerns about the failures of drug prohibition [36], AHR can similarly be framed as a pragmatic and ethical response to the failures of commercialisation.

## 4 Beyond Formal Policy

Beyond co‐ordinated AHR efforts formalised in policy, AHR already occurs in practice across multiple settings. In acute clinical contexts, doctors regularly engage in AHR by negotiating drinking goals, providing preventative supplements such as thiamine, reducing consumption of non‐beverage alcohol and ensuring consumption occurs in safe spaces [37, 38]. In schools, education programs that emphasise recognising and responding to potential alcohol‐related harms have demonstrated effectiveness in reducing harm [39, 40]. In nightlife settings, where there is growing interest in reconciling public health objectives with the sustainability of the hospitality sector [41], AHR has often centred on shared responsibility between peers, venues and communities to prevent intoxication‐related harms [19, 33]. These examples highlight how AHR practices are not absent, but may be fragmented, less systematically evaluated than other interventions, and not clearly articulated within formal policy frameworks.

## 5 Conclusion

Harm reduction is an approach from drug policy with a distinct philosophy and values that is underutilised in alcohol policy. Rather than simply including all policy measures that might reduce harm, it offers a specific focus on reaching marginalised groups, improving service access and improving safer consumption practices. It is typically more complex in implementation and feasibility than consumption‐reduction strategies, and does not address the underlying commercial or social determinants of health. However, AHR can address the temporal and reach limitations of population‐level approaches for those already facing the ‘pointy end’ of harms. While its function has been less clearly articulated and coherently mobilised as a policy strategy, AHR offers ethical and pragmatic interventions that have a growing evidence base [18]. Future research and policy efforts might clarify how harm reduction can be more explicitly integrated within alcohol policy frameworks to best complement population‐level approaches.

## Funding

The author has nothing to report.

## Conflicts of Interest

The author declares no conflicts of interest.
